# USB1 is a miRNA deadenylase that regulates hematopoietic development

**DOI:** 10.1126/science.abj8379

**Published:** 2023-03-02

**Authors:** Ho-Chang Jeong, Siddharth Shukla, Wilson Chun Fok, Thao Ngoc Huynh, Luis Francisco Zirnberger Batista, Roy Parker

**Affiliations:** 1 Division of Hematology, Department of Medicine, Washington University in St. Louis, St. Louis, MO 63110, USA.; 2 Center for Genome Integrity, Siteman Cancer Center, Washington University in St. Louis, St. Louis, MO 63110, USA; 3 Department of Biochemistry, University of Colorado, Boulder, CO 80303, USA.; 4 Howard Hughes Medical Institute, Chevy Chase MD 20815, USA.

## Abstract

Mutations in the 3’ to 5’ RNA exonuclease USB1 cause hematopoietic failure in Poikiloderma with Neutropenia (PN). While USB1 is known to regulate U6 snRNA maturation, the molecular mechanism of PN remains unknown, as pre-mRNA splicing is unaffected in patients. We generated human embryonic stem cells harboring the PN-associated mutation c.531_delA in USB1 and show that this mutation severely impairs human hematopoietic development. We demonstrate that dysregulated miRNA levels in USB1 mutants contributes to hematopoietic failure, due to a failure to remove 3’ end adenylated tails added by PAPD5/7. Modulation of miRNA 3’ end adenylation through genetic or chemical inhibition of PAPD5/7 rescues hematopoiesis in USB1 mutants. This work shows that USB1 can act as a miRNA deadenylase and suggests PAPD5/7 inhibition as a potential therapy for PN.

## One-Sentence Summary

Disease-associated mutations in USB1 affect miRNAs important for hematopoiesis.

Poikiloderma with neutropenia (PN) is an autosomal-recessive bone marrow failure (BMF) syndrome with marked clinical overlap with dyskeratosis congenita (DC) (1). However, unlike patients with DC, telomeres are not significantly shortened in patients suffering from PN, providing a distinguishable feature for the correct diagnosis of PN (2). PN patients harbor homozygous or compound heterozygous mutations in the human gene *C16orf57*, which encodes the conserved 3’ to 5’ RNA exonuclease U6 biogenesis 1 (USB1) (2–5). USB1 is required for the processing of U6 and U6atac snRNAs, and some splicing defects are observed using *in vitro* and zebrafish models of USB1 deficiency (6–10). However, lymphoblastoid cells from PN patients do not exhibit reduced U6 snRNA levels and have normal pre-mRNA splicing (8). These results establish USB1-mediated PN as a singular BMF syndrome, where the underlying genetic cause has been identified but the molecular mechanisms leading to tissue failure are unknown.

To investigate the role of USB1 in a physiological context, we utilized CRISPR/Cas9 to create human embryonic stem cells (hESCs) containing a frequently occurring c.531_del_A loss-of-function mutation in the USB1 gene (hereafter referred to as USB1 mutant) (Figures S1A–B). These USB1 mutant hESCs have normal karyotype (Figure S1C), normal growth rate (Figure S1D), are pluripotent (Figure S1E) and display normal telomere length (Figure S1F), indicating that a clinically-relevant USB1 mutation is not deleterious in undifferentiated hESCs.

**Figure 1.**
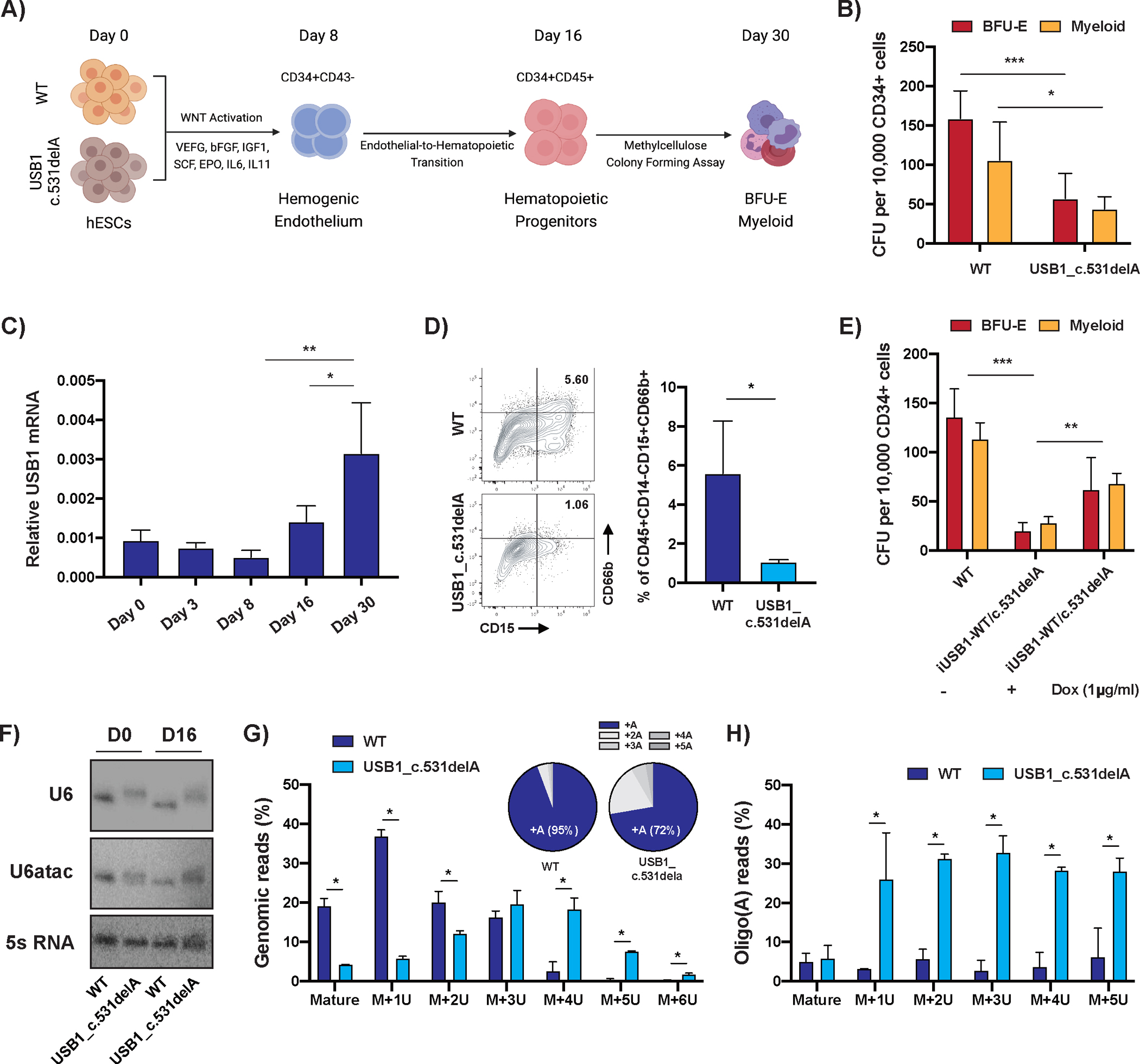
Loss-of-function mutation in USB1 causes hematopoietic impairment. **A)** Model depicting the workflow of hematopoietic differentiation from WT or USB1 c.531delA hESCs. Cellular identity is confirmed by expression of the correct differentiation markers at the different steps depicted in the model. **B)** Colony-forming-cell (CFC) potential of definitive hematopoietic progenitors in WT and USB1 c.531delA cells (average+/−S.D., n=6 biological replicates). **C)** USB1 expression levels analyzed at different stages of hematopoietic specification (average+/−S.D., n=3 biological replicates). **D)** Representative flow cytometry analysis for CD15 and CD66b within CD45+CD14− population on Day 30 of differentiation (left panel). The populations were quantified and graphically presented (right panel, average+/−S.D., n=3 biological replicates). **E)** CFC potential of hematopoietic progenitors in WT and iUSB1-WT/c.531delA cells, treated or not with doxycycline (DOX). Dox (1μg/ml) was added from day 8 to day 16 of differentiation (average+/−S.D., n=3 biological replicates). **F)** Representative northern blot for U6 or U6atac snRNA in either undifferentiated hESCs (D0) or CD34+CD45+ cells (D16) **G) and H)** Bar plots depicting fractions of G) genomic and H) post-transcriptionally adenylated U6 snRNA 3’ ends in WT and USB1 c.531delA cells (Average+/−S.D., n=2 biological replicates). **G – inset:** length distribution of (A) tails at U6 3’ ends in WT and USB1 c.531delA cells.

To elucidate the role of USB1 during hematopoiesis, we performed serum-free hematopoietic differentiations (11–15) to derive hematopoietic progenitor cells from hESCs (Figure 1A). Gene expression analysis confirmed the efficiency of this protocol, with silencing of pluripotency markers and efficient formation of hematopoietic lineages at the end (Day 30) of differentiation (Figure S2A). USB1 mutant cells did not show any impairment during early stages of hematopoietic differentiation, including the formation of mesoderm (Day 3; Figure S2B), and CD34+/CD43− hemogenic endothelium (HE) populations (Day 8; Figure S2C). However, the formation of CD45+ hematopoietic progenitors (Day 16) was decreased in USB1 mutant cells compared to WT cells (Figure S2D), and hematopoietic colony potential analysis showed compromised colony formation in USB1 mutant cells (Figure 1B). Consistent with a role of USB1 in regulating hematopoiesis, USB1 mRNA levels increased ~3-fold in mature blood cells compared to undifferentiated hESCs (Figure 1C). These observations indicate that loss-of-function mutations in USB1 negatively influence hematopoiesis.

As PN is usually associated with severe non-cyclic neutropenia (1), we analyzed the potential of neutrophil formation in WT and USB1 mutant cells. USB1 mutants have reduced formation of CD15+/CD66b+ lineages, indicating abnormal neutrophil development (Figures 1D and S2E). The conditional expression of the WT USB1 protein in USB1 mutants using a Dox-inducible system (Figure S2F) rescued the hematopoietic potential of these cells (Figures 1E). These results recapitulate major clinical manifestations of USB1 deficiency and establish USB1 as an important regulator of hematopoiesis.

To determine the mechanism by which USB1 regulates hematopoiesis, we initially examined if the USB1 mutation affected U6 snRNA. Northern blot analysis of WT and USB1 mutant cells at undifferentiated (D0) and hematopoietic progenitor (D16) stages showed no reduction in the levels of U6 and U6atac snRNAs in USB1 mutants (Figures 1F and S2G–H). However, we observed that U6 and U6atac snRNA from USB1 mutant cells were slightly longer compared to WT cells (Figure 1F), indicating aberrant post-transcriptional processing of these snRNAs similar to what is observed in patient-derived cells (8).

Sequencing the 3’ end of U6 snRNA from WT and USB1 mutant cells revealed two changes. First, while ~40% of all U6 reads terminated at the +1U adjacent to the Lsm 2–8 boundary site in WT cells, in USB1 mutant cells, additional Us were present with the majority of ends terminating at the +3U and +4U (Day 0: Figure 1G; Day 16: Figure S3A). Similar results were observed for the U6atac snRNA (Figure S3B). Second, we observed that all extended U6 and U6atac 3’ ends were oligoadenylated in USB1 mutant cells (Figures 1H and S3C–D), in contrast to approximately 90% of 3’ ends of U6 being monoadenylated in WT cells (Figure 1G – inset; Figures S3E–F). This suggests that the U6 3’ ends are oligoadenylated during U6 snRNA maturation and require USB1 for deadenylation and trimming of the uridylated tail. However, USB1 mutant cells have similar levels of these snRNAs compared to WT cells (Figures 1F and S2G–H) and do not exhibit global pre-mRNA splicing changes (see below), suggesting that USB1 is altering other RNAs to affect hematopoiesis.

**Figure 2.**
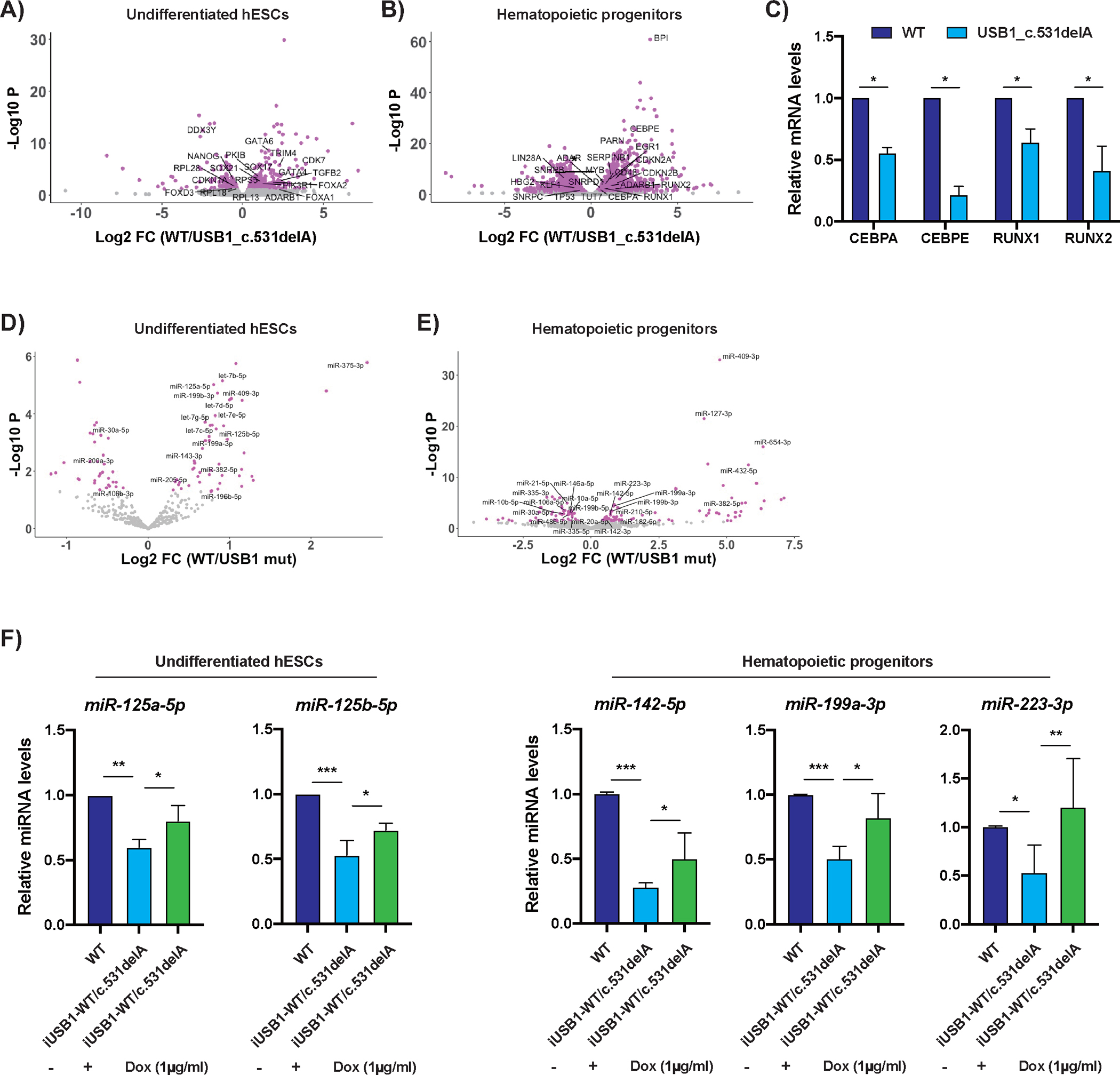
USB1 mutation affects both mRNA and miRNA levels during hematopoietic differentiation. **A) and B)** Volcano plots depicting transcriptome changes in A) undifferentiated hESCs and B) CD34+CD45+ hematopoietic progenitors in WT and USB1 c.531delA cells (Gray: unchanged; Magenta: differentially expressed). **C)** Bar plot depicting levels of indicated mRNAs normalized to 5s rRNA in CD34+CD45+ hematopoietic progenitors (average+/−S.D., n=3 biological replicates). **D) and E)** Volcano plots depicting miRNA changes in D) undifferentiated hESCs and E) CD34+CD45+ hematopoietic progenitors in WT and USB1 c.531delA cells (Gray: unchanged; Magenta: differentially expressed). **F)** Bar plot depicting levels of indicated miRNAs as quantified by Mir-X qRT-PCR normalized to 5s rRNA in both undifferentiated hESCs and hematopoietic progenitors of iUSB1-WT/c.531delA (average+/−S.D., n=3 biological replicates).

To identify other RNAs affected by USB1, we sequenced the transcriptome and miRome of WT and USB1 mutant cells in the undifferentiated, hematopoietic progenitor (CD34+/CD45+) and mature blood cell populations (Figure 1A). We observed few significant changes in gene expression in USB1 mutants in undifferentiated hESCs (469 genes out of 15913 genes were affected with FDR<0.1; 164 genes downregulated more than 2-fold and 61 genes upregulated more than 2-fold) (Figure 2A). As differentiation progressed, we observed more changes in CD34+/CD45+ cells, suggesting that the defect in hematopoiesis occurs during specific stages of differentiation (3310 genes out of 15559 genes were affected with FDR<0.1; 704 genes downregulated more than 2-fold and 577 genes upregulated more than 2-fold) (Figure 2B). Differentially expressed genes in USB1 mutant cells enriched for gene ontology (GO) pathways involved in regulating cell death and neutrophil differentiation (Figure S4A). Consistent with an effect of USB1 deficiency on neutrophil differentiation (Figure 1D), we observed a decrease in high granular SSC populations in USB1 mutant cells upon direct neutrophil development, when compared to WT cells (Figure S4B). Finally, we also observed more gene expression changes in the USB1 mutant cells when compared to WT cells in their mature blood population (2,666 out of 16,310 genes were affected with FDR<0.1; 510 genes downregulated more than 2-fold and 883 genes upregulated more than 2-fold) (Figure S4C). Taken together, this suggests that mutations in USB1 have a greater effect on gene expression in differentiating hematopoietic progenitors and mature blood cells, which correlates with an increase in USB1 levels at these specific stages of differentiation (Figure 1C).

An important result was that we did not detect global splicing changes in the transcriptome of USB1 mutants at any stage when compared to WT cells, and very few differentially expressed genes were mis-spliced in USB1 mutant cells (Figures S4D–E). Previous analysis of PN patient lymphoblasts also did not reveal global splicing changes (8). This is consistent with normal levels of U6 snRNA and U6atac snRNA in USB1 mutant cells and suggests that USB1 deficiency affects gene expression through a mechanism distinct from pre-mRNA splicing. Next, we investigated whether specific mRNA changes in USB1 mutant cells could explain the hematopoietic failure observed, and identified that transcription factors associated with efficient neutrophil formation (i.e. *CEBPA* and *CEBPE*) and overall hematopoiesis (i.e. *RUNX1*, *RUNX2*) are downregulated in USB1 mutants (Figure 2C) (16–19). Combined, these results indicate that despite not affecting U6 levels and pre-mRNA splicing, mutations in USB1 impair correct activation of key hematopoietic and neutrophil development pathways.

We investigated if mutations in USB1 affected miRNA levels in hESCs and their hematopoietic progeny. We observed that the USB1 mutation affected the levels of several miRNAs in undifferentiated hESCs (82 out of 374 miRNAs were affected with p<0.05; 49 miRNAs were downregulated and 33 miRNAs were upregulated) (Figure 2D). We observed increased changes in miRNA levels in CD34+/CD45+ hematopoietic cells in USB1 mutants (131 out of 771 miRNAs were affected with p<0.05; 82 miRNAs were downregulated and 49 miRNAs were upregulated) (Figure 2E). KEGG analysis showed that differentially expressed miRNAs in USB1 mutant cells predominantly affect pathways involved in cancer progression, including acute myeloid leukemia, which is frequently associated with PN (Figure S4F) (20). We verified the downregulation of specific miRNAs in USB1 mutant cells and found that miR-125a-5p, miR-125b-5p, miR-142-5p, miR-199a-3p, and miR-223-3p [which are predominately involved in erythroid, myeloid, and granulocytic differentiation (21, 22)] were decreased in USB1 mutant cells compared to WT cells at different stages of hematopoietic development (Figure 2F). Expression of the WT USB1 protein using a Dox-inducible system rescued the levels of these different miRNAs during different stages of hematopoietic development (Figure 2F). In contrast to changes in miRNA and mRNA levels, we observed few significant changes in other non-coding RNAs (ncRNAs) in undifferentiated (Figures S4G–H) or CD34+/CD45+ (Figure S4I) USB1 mutant cells. These data suggest that USB1 regulates hematopoietic development via miRNA homeostasis, although we cannot rule out the formal hypothesis that other types of ncRNAs might also be involved.

We hypothesized that USB1 might regulate miRNAs by removing 3’ end adenylated tails that would otherwise trigger miRNA degradation for three reasons. First, 3’ oligoadenylation of miRNAs by the non-canonical poly(A) polymerases PAPD5 & PAPD7 can promote their degradation by the cytoplasmic 3’ to 5’ exonucleases DIS3L and/or DIS3L2 (23). Second, removal of oligo(A) tails by the poly(A) specific nuclease PARN stabilizes miRNAs (23). Finally, USB1, while generally thought to act on U tails, can remove poly(A) tails *in vitro* (24). This hypothesis predicts that miRNAs regulated by USB1 would show increased levels of 3’ adenylation in USB1 mutant cells, and that recombinant USB1 protein would deadenylate 3’ adenylated miRNAs. To test this hypothesis, we initially sequenced the 3’ ends of four miRNAs that were reduced in USB1 mutant cells and known to regulate hematopoiesis (21, 22).

**Figure 3.**
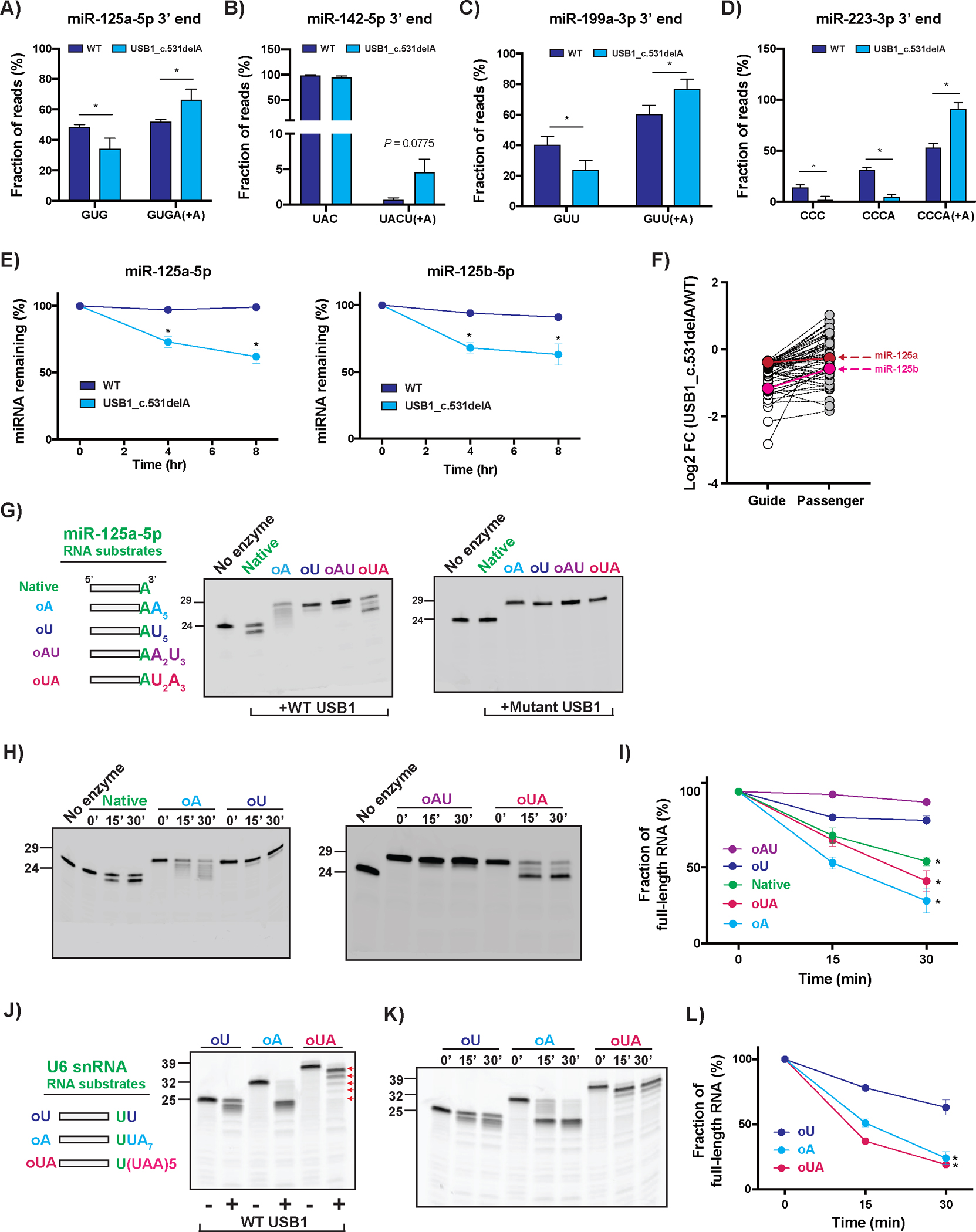
USB1 deadenylates RNA substrates *in vivo* and *in vitro*. **A)** Bar plot depicting fraction of genomic and post-transcriptionally adenylated miR-125a-5p 3’ ends in undifferentiated WT and USB1 c.531delA cells (average+/−S.D., n=2 biological replicates). **B-D)** Bar plots depicting fraction of genomic and post-transcriptionally adenylated B) miR-142-5p, C) miR-199a-3p, and D) miR-223-3p 3’ ends in CD34+CD45+ hematopoietic progenitors in WT and USB1 c.531delA cells (average+/−S.D., n=3 biological replicates). **E)** Quantification of miR-125a-5p or miR-125b-5p decay rates in WT and USB1 c.531delA at 0, 4, and 8hr after transcription shutoff (average+/−S.D., n=3 biological replicates). **F)** Influence of USB1 on miRNA guide and passenger strands. Shown are the expression levels of guide and passenger strands for miRNAs with the highest differential expression between USB1 c.531delA mutants and WT hESCs (average, n=3 biological replicates). **G)** Representative gel images showing processing of miR-125a-5p substrates by wild-type USB1 or H208Q catalytic mutant. miR-125a-5p sequence includes a genomically encoded A at the 3’ end (shown in green). Marker for 24-nt indicates a native form of miR-125a-5p. **H)** Representative gel images depicting time-course measurement of USB1’s activity on indicated miR-125a-5p RNA substrates. **I)** Line plot depicting degradation of miR-125a-5p RNA substrates with indicated 3’ end modifications (average+/−S.D., n=3 technical replicates). **J)** Representative gel image showing processing of U6 snRNA substrates by wild-type USB1. Genomically encoded bases are shown. Red arrows show position of trimmed UAA intermediates for the oUA substrate incubated with wild-type USB1. **K)** Representative gel image depicting time-course measurement of USB1’s activity on indicated U6 snRNA substrates. **L)** Line plot depicting degradation of U6 snRNA substrates with indicated 3’ end modifications (average+/−S.D., n=3 technical replicates).

Strikingly, the USB1 mutation led to an increase in the levels of adenylated reads at 3’ ends of miR-125a-5p in undifferentiated hESCs (Figure 3A). In WT cells, miR-125a-5p terminates at either the genomically encoded 5’-GUG-3’ or at 5’-GUGA-3’ in a 1:1 ratio (Figure 3A). However, in USB1 mutant cells, we observed a decrease in the 5’-GUG-3’ fraction and a proportional increase in the 5’-GUGA-3’ fraction (Figure 3A). Similar results were observed for miR-142-5p, miR-199a-3p and miR-223-3p in CD34+/CD45+ hematopoietic progenitors, with USB1 mutants containing more adenylated ends when compared to WT (Figures 3B–D). Broader analysis of miRNA-sequencing in WT and USB1 mutant hESCs (Figure S5A), or in WT and K562 hematopoietic cells where we ablated USB1 (USB1-KO; Figure S5B), demonstrated that most miRNAs with reduced expression levels show increased 3’ adenylation in USB1 mutants, either in hESCs or K562s. A possibility that at this point cannot be discarded is that USB1 might also increase levels of some miRNAs by steric inhibition of nucleases, and/or by creating a 2’–3’ cyclic phosphate at the 3’ end of miRNAs, a USB1-dependent modification shown in the U6 snRNA (8, 25).

Two observations argue USB1 affects the decay rate of some miRNAs. First, following transcriptional inhibition with actinomycin D, USB1 mutant hESCs show increased rates of decay of miR-125a-5p and miR-125b-5p (Figure 3E) and miR-142-5p, miR-199a-3p, and miR-223-3p (Figure S5C). These results were further confirmed when we compared miRNA decay rates in WT and USB1-KO K562 hematopoietic cells (Figure S5D). Second, although guide miRNA strands decreased, we observed that levels of passenger miRNA strands remained similar in WT and USB1 mutants (Figure S5C). These results were also observed in K562 cells (Figure S5E), and further confirmed by miRNA sequencing analysis (Figure S6A), which shows that at a global level, USB1 impairment mostly affects guide, and not passenger miRNA strands (Figure 3F: hESCs; Figure S6B: K562).

To test if USB1 could directly deadenylate an adenylated miRNA, we purified recombinant human USB1 (wild-type and a catalytically inactive mutant H208Q) and tested its activity on 5’-FAM labeled miR-125a-5p substrates with different 3’ end additions (Figure 3G). We observed that wild type, but not catalytically inactive USB1, efficiently removed the adenosine(s) from the 3’ end of miR-125a-5p in a time-dependent manner, as seen by the shortened products observed for native miR-125a-5p (which has a single A at its 3’ end), oA and oUA substrates (Figures 3G–I). USB1 was also able to rapidly remove adenosines from the 3’ end of an U6 snRNA oligo (Figures 3J–L). This demonstrates USB1 can function as a deadenylase for miRNAs and U6 snRNAs *in vitro*.

Our data suggests that USB1 can also remove single uridine residues from RNA substrates but cannot efficiently remove poly(U) tails. Specifically, USB1 slowly removed one uridine from the 3’ end of oligouridylated miR-125a-5p substrates, as observed by the appearance of a small amount of product shorter by one nucleotide (Figures 3G–I), which we confirmed with shorter RNA substrates (Figures S6C–D). USB1 also removed a single uridine residue from an oligouridylated 3’ end of the U6 snRNA (Figures 3J–K).

**Figure 4.**
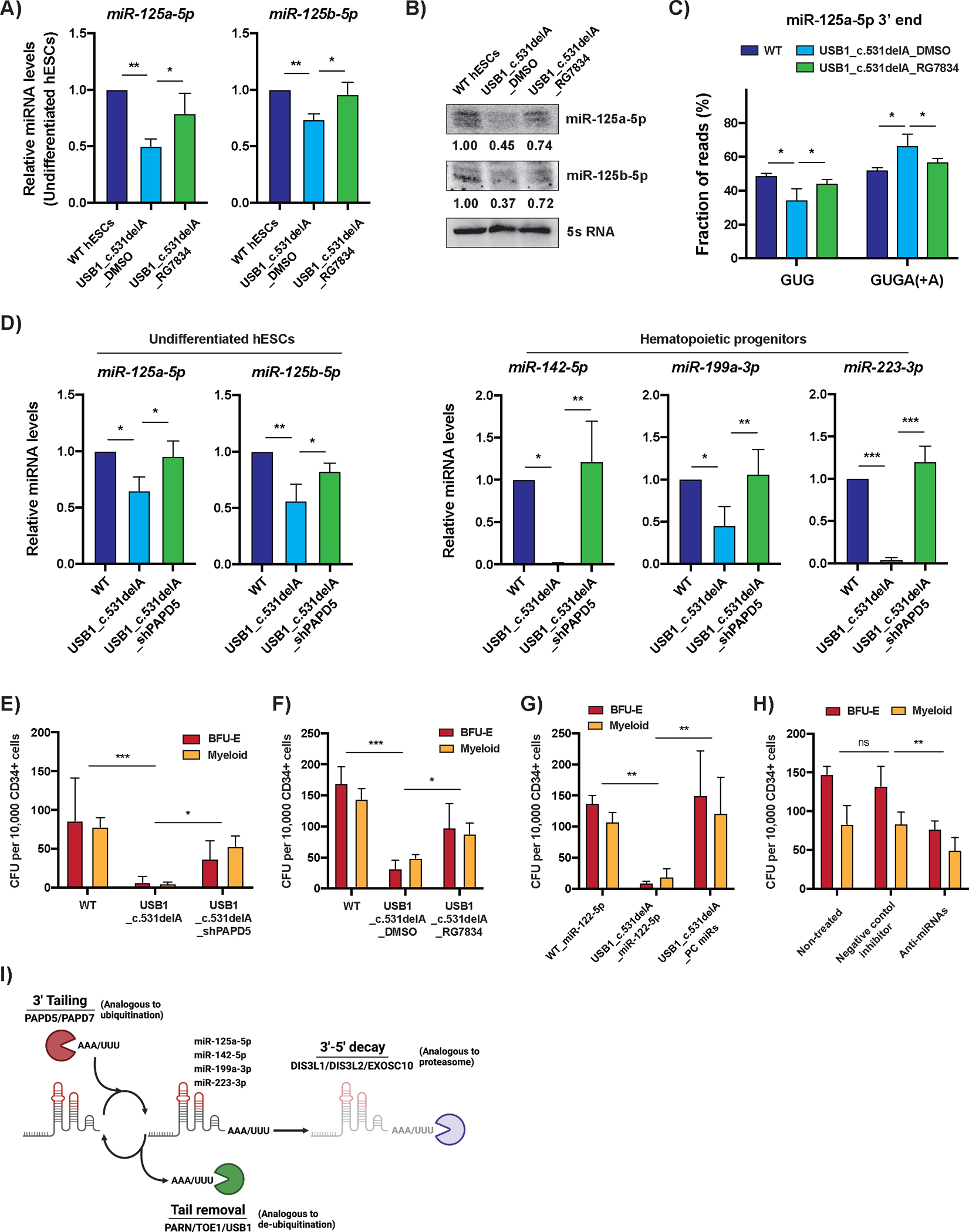
Inhibition of PAPD5/7 and modulation of miRNA levels rescues hematopoietic output in USB1 mutants. **A)** Bar plot depicting levels of indicated miRNAs normalized to 5s rRNA in undifferentiated USB1 c.531delA cells treated with DMSO or RG7834 (1μM) (average+/−S.D., n=3 biological replicates). **B)** Representative northern blot analysis for miR-125a-5p and miR-125b-5p in undifferentiated USB1 c.531delA cells treated with DMSO or RG7834 (1μM). Relative band intensity of indicated miRNAs normalized to 5s rRNA is shown (n=2 biological replicates). **C)** Bar plot depicting fraction of genomic and post-transcriptionally adenylated miR-125a-5p 3’ ends in undifferentiated USB1 c.531delA cells treated with RG7834 (1μM) (average+/−S.D., n=2 biological replicates). **D)** Bar plots depicting indicated miRNAs (quantified by Mir-X qRT-PCR) normalized to 5s rRNA in both undifferentiated hESCs and hematopoietic progenitors in USB1 c.531delA_shPAPD5 cells (average+/−S.D., n=3 biological replicates). **E)** CFC potential of definitive hematopoietic progenitors in WT, USB1 c.531delA and USB1 c.531delA_shPAPD5 cells (average+/−S.D., n=3 biological replicates). **F)** CFC potential of definitive hematopoietic progenitors in WT and USB1 c.531delA treated with DMSO or RG7834 (average+/−S.D., n=3 biological replicates). RG7834 (0.5μM) was added from day 8 to day 16 of differentiation every other day. **G)** CFC potential of definitive hematopoietic progenitors in WT_miR-122-5p, USB1_miR-122-5p and USB1 c.531delA_PC miRs. PC miRs indicate the polycistronic expression of miR-125a-5p, 142-5p, 199a-3p, and 223-3p. The expression of miR-122-5p in WT and USB1 mutant cells was performed as a control (average+/−S.D., n=3 biological replicates). **H)** CFC potential of definitive hematopoietic progenitors in WT cells treated with miRNA inhibitors (Anti-miRs) specifically targeting miR-125a-5p, 142-5p, 199a-3p, and 223-3p. miRNA inhibitors (20nM) were added from day 8 to day 16 of differentiation (average+/−S.D., n=4 biological replicates). **I)** Model depicting the regulation of non-coding RNA stability through competition between ‘Tailing’ and ‘Tail removing’ enzymes. This system is analogous to the ubiquitin-mediated proteasome degradation system, where RNAs are tagged for degradation by 3’ end modification by enzymes such as PAPD5 and PAPD7, and protector exonucleases such as PARN and USB1 remove the post-transcriptional modifications to stabilize the RNA. In the absence of tail removal, the 3’ end modified RNA would be degraded by 3’ to 5’ exonucleases such as EXOSC10.

Our data suggests that a loss of expression of different miRNAs required for blood development contributes to the hematopoietic deficit observed in PN patients due to the failure of USB1 to remove adenylated tails added by PAPD5/7. Accordingly, we observed that inhibiting PAPD5 with RG7834, an inhibitor of PAPD5/7 non-canonical poly(A) polymerases (26), rescued levels of miRNAs that were reduced in USB1 mutant cells (Figures 4A–B). Moreover, treatment of USB1 mutants with RG7834 led to a decrease in adenylated miR-125a-5p, which was compensated by an increase in the non-adenylated form, demonstrating that these enzymes are responsible for adding adenylated tails to the 3’ end of miRNAs that are increased in USB1 mutants (Figure 4C). Inhibition of PAPD5 by constitutive silencing using shRNAs (Figure S7A) also rescued the levels of miRNAs affected by USB1 mutation to approximately WT levels (Figure 4D). These results suggested that inhibition of PAPD5/7 activity should rescue the hematopoietic development defects seen in USB1 mutant cells by preventing the 3’ end adenylation of miRNAs. To test this hypothesis, we investigated if the genetic silencing of PAPD5 or treatment with RG7834 could rescue hematopoietic differentiation of USB1 mutants.

We observed that the compromised colony-formation potential in USB1 mutants is rescued by both the genetic (Figure 4E) and chemical (Figure 4F) inhibition of PAPD5. The chemical inhibition of PAPD5/7 by RG7834 treatment in WT cells does not affect miRNA levels (Figure S7B) or colony-forming potential (Figure S7C), consistent with our previous report (14). Importantly, RG7834 treatment also improved neutrophil formation in USB1 mutant cells, a key feature of hematopoietic failure in PN (Figure S7D). These results indicate that inhibition of PAPD5/PAPD7 could be a potential therapeutic strategy for treating these patients. Treatment with RG7834 did not rescue the extended U6 3’ end (Figure S7E), giving further support to hematopoietic failure in PN being independent from U6.

To directly test if the miRNAs downregulated in USB1 mutants are responsible for the hematopoietic failure observed, we engineered USB1_c.531delA mutant hESCs with constitutive expression of miR-125a-5p, miR-142-5p, miR-199a-3p, and miR-223-3p (Figure S7F). This led to a rescue of both erythroid and myeloid hematopoietic outputs in USB1 mutants, to levels similar to WT cells (Figure 4G). This demonstrates miRNA deficiencies are linked to hematopoietic failure in USB1 mutants. Moreover, treating WT CD34+ hematopoietic cells with miRNA inhibitors targeting miR-125a-5p, miR-142-5p, miR-199a-3p, and miR-223-3p (Figure S7G), caused a significant decline in their colony forming ability (Figure 4H). These results demonstrate that the hematopoietic failure observed in USB1 mutants is directly linked to impaired levels of these miRNAs. This conclusion is corroborated by the role of Dicer and Ago-2 in blood cell development (27–29).

In conclusion, we showed that USB1 functions to deadenylate miRNAs, limiting their degradation and increasing their abundance, and this deadenylating activity of USB1 regulates hematopoiesis. This identifies USB1 as a second de-tailing enzyme, which similar to PARN (23), can remove oligo(A) tails from ncRNAs to enhance their stability (Figure 4I). Importantly, inhibition of the enzymes responsible for miRNA adenylation, PAPD5 & PAPD7, rescued the hematopoietic deficit observed in the USB1 mutant cells. Our results provide a molecular understanding of the pathogenesis observed in patients with USB1 mutations and suggest that PAPD5/7 inhibitors might be a treatment option for PN patients.

## Supplementary Material

Supplemental Material

## Data Availability

All data are available in the main text or the supplementary materials. Raw sequencing data for RNA-seq and miRNA-seq will be available at a public repository before publication.
