# Supplementary material for: USB1 is a miRNA deadenylase that regulates hematopoietic development
Source: Science. Author manuscript; Available in PMC 2024 Jan 30. (PMC10827040; doi:10.1126/science.abj8379)
Supplement: Supplemental Material [file NIHMS1957524-supplement-Supplemental_Material.docx]

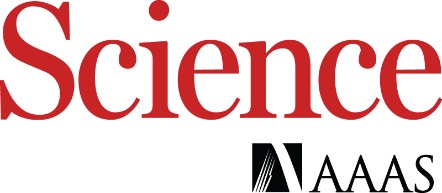


Supplementary Materials for

USB1 is a miRNA deadenylase that regulates hematopoietic development

Ho-Chang Jeong^1,2^†, Siddharth Shukla^3,4^†, Wilson Chun Fok^1,2^, Thao Ngoc Huynh^3,4^, Luis Francisco Zirnberger Batista^1,2^*, and Roy Parker^3,4^*

Correspondence to: lbatista@wustl.edu; roy.parker@colorado.edu

**This PDF file includes:**

Materials and Methods

Figs. S1 to S7

Table S1 to S3

Materials and Methods

**Cell culture**

H1 hESCs (WA01) were acquired from WiCell Research Institute (Madison, WI), following all institutional guidelines determined by the Embryonic Stem Cell Research Oversight Committee (ESCRO) at Washington University in St. Louis. hESCs were maintained in mTeSR1 medium (StemCell Technologies, Vancouver, Canada) on plate coated with Matrigel (BD Biosciences, San Jose, CA) diluted in DMEM/F12 supplemented with 1% Pen/Strep. For feeder-based conditions, hESC basal medium (DMEM/F12 supplemented with 1% Non-essential amino acids, 1% 2-mercaptoethanol, and 1% Pen/Strep) with 20% KnockOut Serum Replacement, 1X L-glutamine, and 10 ng/ml bFGF was used on growth-arrested mouse embryonic fibroblasts (MEFs). hESCs were cultured in a humidified incubator at 37^o^C in 5% CO_2_ and 5% O_2_ levels. For K562, cells were cultured in Iscove’s Modified Dulbecco’s Medium, supplemented with 10% Fetal Bovine Serum and 1% Pen/Strep at 37^o^C in 5% CO_2_ incubator.

**Gene editing**

USB1_c.531delA hESCs were generated using CRISPR/Cas9 genome-editing technology. Briefly, CRISPR gRNAs were inserted into the MLM3636 plasmid (Addgene #43860) and transfected with Cas9 plasmid (Addgene #43945) and single-stranded DNA donor oligos using Lonza’s 4D-Nucleofector with the P4 Primary Cell 4D-Necleofector kit (Allendale, NJ). Cells were seeded at low density, picked manually, and then sequenced. Donor oligo sequences were used as follows; 5’-ATTGGGCTTGAGGTCACTTC-NGG-3’ and 5’-TTGGGCTTGAGGTCACTTCA-NGG-3’. iUSB1-WT/c.531delA and USB1_c.531delA_shPAPD5 hESCs were engineered by zinc finger nuclease by targeting the AAVS1 locus. Transfection was performed with X-TremeGene 9 transfection kit (#06 365 779 001; Roche) following the manufacturer’s instructions. For shPAPD5, three shRNA hairpins obtained from the Broad Institute were inserted into MIR30 cassette of the AAVS1-GAG-GFP-mir30 plasmid containing AAVS1 homology regions. The shRNA sequences were used as follows; 5’-GCCACATATAGAGATTGGATA-3’, 5’-CGATGTTGGAAGGAGTTCATA-3’, 5’-CCCAATACAAACTATGGTGTT-3’.

**USB1 deletion in K562 hematopoietic cells**

USB1-targeting sgRNAs were designed using CHOPCHOP (https://chopchop.cbu.uib.no/) and oligos were cloned into PX459 Cas9-sgRNA expression plasmids (Addgene 62988). 10^6^ K562 cells were suspended in 500μl of OPTI-MEM, nucleofected (20μg of sgRNAs), and selected (10μg/ml of puromycin) for 48 hr after nucleofection. Surviving clones were serially diluted as single cells in 96 well plates. Each single clone was expanded in selection medium and screened for loss of USB1 through immunoblotting. sgRNA sequence: 5’-ACCTTCATCCTTACCAGCCG-3’.

**Lentiviral transduction**

To establish USB1_c.531delA hESCs with constitutive expression of miR-125a-5p, 142-5p, 199a-3p, and 223-3p, polycistronic (PC) lentiviruses expressing target miRNAs were produced by transfecting 293T cells with third-generation lentivirus packaging plasmids (pMDL [Gag/Pol], pVSVG, and pREV). miR-122-5p viruses (used as negative control) were produced following the same protocol. Plasmids were synthesized by Vector Builder. Supernatants were collected at 48 hr post-transfection, filtered (0.45 µM), concentrated with Lenti-X Concentrator (Takara), and transduced into WT or USB1_c.531delA hESCs (two rounds of transduction were performed). EGFP-positive hESCs were sorted on BD FACS Aria-II at the Department of Pathology & Immunology Flow Cytometry Core (Washington University in St. Louis).

**Hematopoietic differentiation**

Definitive hematopoietic differentiation from hESCs was performed as previously described (*11, 12*). Briefly, hESCs were cultured on growth factor reduced matrigel (BD Bioscience) for 1 day to deplete MEFs. On Day 0, cells were dissociated with 0.05% trypsin (Gibco) for 1 min and incubated with serum-free media [SFD: IMDM supplemented with 25% Hams F12, 0.05% BSA, 1X B27 supplement (Gibco), 0.5X N2 supplement (Gibco), 2mM L-glutamine (Gibco), 50µg/ml ascorbic acid (Gibco), 4x10^-4^ M monothioglycerol (Millipore Sigma), and 150µg/ml transferrin] supplemented with 10ng/ml BMP4 on 6 well plate coated with a 5% poly(2-hydroxyethyl methacrylate) solution (Millipore Sigma). On Day 1, one additional volume of SFD was added, containing 10ng/ml BMP4 and 5ng/ml bFGF. On Day 2, media was changed to fresh SFD supplemented with 10ng/ml BMP4, 5ng/ml bFGF, 3µM CHIR99021, and 6µM SB-431542. On Day 3, media was changed to StemPro-34 media [SP-34: StemPro-34 supplemented with 2mM L-glutamine (Gibco), 1mM ascorbic acid (Gibco), 4x10^-4^M monothioglycerol, 150µg/ml transferrin] supplemented with 15ng/ml VEGF and 5ng/ml bFGF. On Day 6, one additional volume of SP-34 supplemented with 15ng/ml VEGF, 5ng/ml bFGF, 20ng/ml IL-6, 50ng/ml IGF1, 10ng/ml IL-11, 200ng/ml SCF, and 4IU EPO was added. For primitive hematopoietic differentiation, on Day 2, the media was changed to fresh SFD supplemented with 10ng/ml BMP4, 5ng/ml bFGF, 1ng/ml Activin A, and 3µM IWP2. Every step was maintained in a 5% CO_2_ and 5% O_2_ incubator. All cytokines were purchased from R&D BioSystems (Minneapolis, MN), but EPO and IGF1 were obtained from Peprotech (Rocky Hill, NJ).

**Methylcellulose assays and Neutrophil development**

Colony forming cell (CFC) assay was performed using MethoCult H4034 Optimum (StemCell Technologies, Vancouver, Canada). 10,000 CD34+CD43- or CD34+CD43-CD73-CD184- cells sorted at Day 8 were aggregated overnight in a well of a 96 well low-adhesion plate at 2x10^5^ cells/ml density in 50µl of SP-34 supplemented with 30ng/ml TPO, 30ng/ml IL-3, 100ng/ml SCF, 10ng/ml IL-6, 5ng/ml IL-11, 25 ng/ml IGF1, 2IU EPO, 5ng/ml VEGF, 5ng/ml bFGF, 10ng/ml BMP4, 10ng/ml FLT3L, and 20ng/ml SHH. Aggregates were transferred to a well of a Matrigel-coated 24 well plate and 1ml of the same supplemented SP-34 media was added 6 hr post-transfer. Cells were maintained in 5% CO_2_ and 5% O_2_ incubator for 8 days (Day 16) and then placed into 1ml of MethoCult in which the colonies forming were measured after 12 days. For neutrophil development, cells from Day 16 were further incubated with RPMI medium containing 10% FBS, and 100ng/ml G-CSF for additional 10 days as described previously (*30*). Blood counts from high SSC populations were performed using a HEMAVET multispecies hematology analyzer (Drew Scientific). All cytokines were purchased from R&D BioSystems, but EPO and IGF1 were obtained from Peprotech.

**Transfection of miRNA inhibitors**

miRIDIAN microRNA hairpin inhibitors were added (20nM) to hematopoietic differentiation cultures on Day 8 and Day 12 in 24 well plates (10^4^ cells/well), using 1μl/well of DharmaFECT-1 transfection reagent (Dharmacon) according to the manufacturer’s instructions. miRIDIAN microRNA hairpin inhibitors for miR-125a-5p (IH-300624-06), 142-5p (IH-300609-06), 199a-3p (IH-300535-06), or 223-3p (IH-300580-08) and negative control #1 (IN-001005-01) were purchased from Dharmacon.

**Flow cytometry and Cell sorting**

Flow cytometry analysis was performed using BD X20 and cell sorting was done on BD FACS Aria-II at the Department of Pathology & Immunology Flow Cytometry Core (Washington University in St. Louis). The antibodies were used as follows; SSEA-4 (clone MC-813-70), TRA-1-81 (clone TRA-1-81), KDR (clone 89106), CD235a-APC (clone HIR2), CD34-APC (clone 8G12), CD34-PE-Cy7 (clone 4H11), CD43-PE or -FITC (clone 1G10), CD73-PE (clone AD2), CD184-APC (clone 12G5), CD45-PE or -BV421 (clone HI30), CD11b-PE (clone D12), CD14-PE-Cy7 (clone MφP9), CD15-BV421 (W6D3), CD16-APC (clone B73.1), CD66b-FITC (clone G10F5). All antibodies were purchased from BD Biosciences, except for CD15 and CD66b (BioLegend) or SSEA-4 and TRA-1-81 (Millipore). For cell cycle analysis, Click-iT EdU Alexa Fluor 488 kit (Life Technologies) was used as following manufacturer’s instructions.

**RNA extraction and Quantitative real-time PCR**

Total RNA was isolated using Trizol (Invitrogen) with Phasemaker tubes in accordance with the manufacturer’s instructions. Contaminating genomic DNA was removed using Turbo DNA-free kit (Invitrogen). RNA concentration was measured using Qubit assay (Thermo Fisher). For the measurement of mRNA levels, cDNA was synthesized from total RNA using RNA-to-cDNA EcoDry premix (Double primed) (Takara Biotech). qRT-PCR was performed using iQ SYBR Green Supermix (BioRad) on the CFX96 thermocycler (BioRad). Relative mRNA levels were calculated using the 2^-ΔΔCt method. For the measurement of miRNA levels, cDNA was synthesized using Mir-X miRNA first strand synthesis kit (Takara Biotech). qRT-PCR was performed using TB Green Advantage qPCR premix (Takara Biotech) on the CFX96 thermocycler (BioRad). The entire miRNA sequence was used as a reverse primer for qRT-PCR and primer sequences for mRNA were listed in Table S1.

**Northern blotting**

Total RNA was extracted as described above. After quantification on Nanodrop, 10 μg of total RNA was separated on an 8% polyacrylamide 7M Urea gel in 1X TBE. RNA was transferred to a Nytran Supercharge blotting membrane (Thermo Fisher). After cross-linking, membranes were pre-hybridized and hybridized in PerfectHyb-Plus Hybridization Buffer (Sigma Aldrich) at 42°C. miRNA LNA probes for each target miRNA were purchased from Exiqon. Probes are described in Table S2. Membranes were then washed (2X SSC 0.1% SDS), exposed to a cassette and imaged on a Typhoon FLA 9500 Phosphoimager. Densitometry analysis was performed using Fiji.

**Transcriptional shut-off experiments**

For measurement of miRNA decay rates, WT and USB1 c.531delA hESCs were treated with actinomycin D (Sigma) at 5µg/ml. Cells were harvested at 0, 4, or 8 hr post-treatment. For WT and USB1-KO K562, cells were treated with actinomycin D at 5µg/ml and harvested at 0, 4, or 7 hr post-treatment. RNA was collected as described above.

**RNA-seq library preparation and Analysis**

For strand-specific library preparation, DNA-free total RNA was used as a starting material. Ribosomal RNA was depleted using RiboCop rRNA depletion kit HMR V2 (Lexogen) as per manufacturer’s protocol. 10 ng of depleted RNA was used as input for library preparation using the NextFlex Rapid Directional RNA-Seq kit 2.0 (Perkin Elmer) as per manufacturer’s protocol. Libraries were analyzed on Bioanalyzer (Agilent) to check for concentration and correct fragment insertion. Libraries were pooled together and sequenced on the NovaSeq 6000 (Illumina) using the 2x150 kit to obtain paired ends RNA-seq reads. Reads were demultiplexed and adapters and low-quality bases were trimmed using Trimmomatic (*31*). Trimmed reads were aligned to the human cDNA transcriptome (ENSEMBL GRCh38 v99) and were quantified using Salmon (*32*). For non-coding RNA abundance quantification, reads were specifically aligned to the human ncRNA transcriptome (ENSEMBL GRCh38 v99) and were quantified using Salmon (*32*). Transcript quantifications were imported to DeSeq2 and differential expression analysis was performed under default parameters (*33*). Downstream analysis and visualization were performed in R. For analysis of splicing differences between WT and USB1 c.531delA at different stages of differentiation, trimmed RNA-seq reads were aligned to the human genome (ENSEMBL GRCh38 v99) using STAR (*34*). Aligned SAM files were coordinate-sorted and compared to BAM. Indexed BAM files were used as input and local splice variations (LSVs) were calculated in each sample using MAJIQ (*35*). Differentially spliced LSVs obtained from MAJIQ were converted to TSVs using Voila tool in the MAJIQ package.

**miRNA-seq library preparation and Analysis**

300 ng of DNA-free total RNA was used as input for library preparation using the Small RNA-seq library prep kit (Lexogen). Amplified libraries were size-selected to enrich for smaller inserts using the companion magnetic bead module. Libraries were pooled together and sequenced on the NovaSeq 6000 (Illumina) using the 2x150 kit to obtain paired ends RNA-seq reads. Reads were demultiplexed and adapters were trimmed using Cutadapt (*36*). Trimmed reads were aligned to human miRNA database (miRbase V22) using Bowtie2 allowing no mismatches in the seed-sequence (*37*). Aligned miRNA counts were extracted from sorted BAM alignments and differential expression analysis was performed using DESeq2 (*33*). Downstream analysis and visualization were performed in R.

**3’ end library preparation and Analysis**

3’ end libraries were prepared as previously described (*23, 38*). Briefly, 30 ng of DNA-free rRNA-depleted RNA was treated with Antarctic Phosphatase (NEB), and two RNA appendices containing a unique barcode were ligated to equal amount of the input RNA using T4 RNA Ligase I (NEB). Reactions were cleaned up using RNA clean & concentrator kit (Zymo Research) and cDNA was prepared using an RNA appendix-specific primer and Superscript III RT (Invitrogen). cDNA was used as a template for 5’ RACE using a miRNA-specific reverse primer containing a barcode, a universal primer and Phusion HF DNA polymerase (NEB). RACE products were separated on a 1% Agarose TAE gel, 100-180 bp DNA fragments were excised and DNA was eluted. Illumina-compatible libraries were prepared by amplifying RACE product using Illumina primers, and sequenced on a HiSeq lane (Illumina) using the 2x150 kit. Libraries were demultiplexed and unique reads aligning to the exact miRNA sequence were quantified. miRNA 3’ ends were obtained from miRbase v22.

**miRNA tail analysis using Tailer**

miRNA read processing and mapping to the miRbase 22 reference was performed as described above. SAM alignment files were used as input into Tailer (*39*) using the “--mirna” flag as described in the documentation. Tail output was miRNA-sorted and reads corresponding to each miRNA end (with and without tails) were summarized. Data for each miRNA is internally normalized to total reads for that particular miRNA and represented as a fraction of all read at any position corresponding to a particular miRNA.

**Recombinant USB1 purification**

Human USB1 cDNA sequence was cloned in the NdeI-XhoI cleavage site of the PColdI E. coli expression vector (Takara Biotech). Catalytic mutant USB1 was prepared by site-directed mutagenesis of the wild-type USB1 plasmid by changing H208 to Q. The plasmid was transformed in Rosetta2 (DE3) cells and USB1 expression was induced using cold shock and 1 mM IPTG as per manufacturer’s instruction (Takara Biotech). The culture was pelleted and lysed by sonication in lysis buffer as described previously (*24*). His-USB1 was purified using Ni-NTA resin (Qiagen) and eluted using 500 mM Imidazole. Eluted protein was concentrated using Amicon filter tubes by centrifugation with 10 kDa cutoff to a 500 μl volume. Concentrated protein was applied to an AKTA FPLC with Superdex 75 increase 30 column (separation range between 7 and 75 KDa) and USB1 was purified using size exclusion chromatography. Fractions were pooled and concentrated down to 1 ml volume in Amicon 10 KDa filter and buffer was exchanged to storage buffer using dialysis. Purified protein was aliquoted and stored at -80^o^C.

**RNA degradation assays with recombinant USB1**

5 μM of USB1 was incubated with 1 μM of RNA substrate in 10 μl reaction volume at 37^o^C for the described duration in 1X reaction buffer (20 mM Hepes pH 7.4, 100 mM NaCl, 1 mM EDTA) as previously described (*24*). Reactions also contained RNAsin (Promega) to prevent contaminating RNAse activity and 1 mM TCEP pH 7.4. Reactions were stopped with 2X formaldehyde loading dye and reaction products were separated on a 15% acrylamide TBE-Urea gel (Invitrogen) in 1X TBE buffer. Fluorescent RNA was visualized on a Typhoon Phosphoimager and densitometry analysis was performed using Fiji. RNA oligos used for the assays containing a 5’-FAM label (Table S3) were purchased from IDT.

**Telomere length analysis**

Telomere length was quantified by Telomere Repeat Fragment (TRF). Briefly, 10 µg of DNA was digested with RSA and HINF1 restriction enzymes (New England Biolabs, Ispwich, MA) overnight and 2.5 µg of product was resolved in a 0.75% agarose gel for 16 hr at 85V in TBE buffer. The gel was incubated with denaturing buffer (1.5M NaCl and 0.5M NaOH) for 45 min followed by neutralizing buffer (1.5M NaCl, 1M Tris-HCl at pH 7.4) for 1 hr. DNA was transferred to a nitrocellulose membrane by capillary action for 2 days in 10X saline-sodium citrate (3M NaCl, 0.3M sodium citrate dehydrate at pH 7.0). After cross-linking, the membrane was hybridized with a ^32^P-labelled probe (TTAAGGG)_4_ and exposed to Carestream BioMax MT film.

**Immunoblotting**

Cells were lysed with NP-40 buffer (25mM Hepes, 150mN KCI, 1.5mM MgCl_2_, 0.5% IGEPAL CA-630, and 10% glycerol; pH 7.5 in RNase-free water) supplemented with Pierce protease inhibitor (Thermo) and PhosSTOP (Roche) following the manufacturer’s instructions. Protein quantification was performed using a BCA protein assay kit (Thermo) and immunoblotting assay was done as described previously (*15*). Primary antibodies used in this study were as follows; USB1 (1:1000, Abcam) and ACTB (1:2000, Sigma).

**G-banded karyotyping**

The karyotypes were determined by Cell Line Genetics (Madison, WI).

**Statistical analysis**

Graphical data are presented as mean ± standard deviation (SD). Statistical significance for more than three groups was determined using one-way or two-way analysis of variance (ANOVA) following a Tukey multiple comparison post-test. Statistical significance between the two groups was analyzed using unpaired Student’s t-tests. The statistical significance was assumed to be ns: not significant, **p* < 0.05, ***p* < 0.01, and ****p* < 0.001.

**Fig. S1.**

**A)** Strategy for introduction of the disease-specific USB1 c.531delA mutation in hESCs. Guide RNA targeting exon 5 of USB1 was used in combination with specific single-strand DNA donor oligo templates. Silent mutations were introduced to facilitate CRISPR/Cas9-mediated genome modification (shown in blue). **B)** Sequencing traces confirming genome modification. **C)** G-band karyotyping in WT and USB1 c.531delA hESCs. **D)** EdU incorporation assay was performed in WT and USB1 c.531delA hESCs. The percentages of active proliferating cells (EdU+) were shown. **E)** Flow cytometry analysis for pluripotency markers in WT and USB1 c.531delA hESCs. **F)** Telomere restriction fragment assays depicting telomere lengths in WT and USB1 c.531delA hESCs over multiple passages.

**Fig. S2.**

**A)** mRNA levels during definitive hematopoietic specification in WT cells (average+/-S.D., n=3 biological replicates). Specific genes are indicated in each panel. **B)** Flow cytometry analysis for CD235a and KDR from Day 3 definitive (CD235a-) hematopoietic differentiation (average+/-S.D., n=4 biological replicates). **C)** Flow cytometry analysis for CD34 and CD43 from Day 8 of definitive hematopoietic differentiation (average+/-S.D., n=5 biological replicates). **D)** Flow cytometry analysis for CD45+ from Day 16 of definitive hematopoietic differentiation (average+/-S.D., n=4 biological replicates). **E)** Flow cytometry analysis for FSC and SSC upon neutrophil development in WT and USB1 c.531delA cells (left panel). High SSSC populations were gated (shown in red). Blood neutrophils were quantified (right panel) using a clinical hematology analyzer (Hemavet). **F)** USB1 mRNA (left panel) or protein (right panel) levels were assessed in iUSB1-WT/c.531delA hESCs upon Dox treatment in a dose-dependent manner (average+/-S.D., n=3 biological replicates). **G) and H)** Bar plots indicate G) U6 and H) U6atac snRNA levels normalized to 5s rRNA (average+/-S.D., n=3 biological replicates).

**Fig. S3.**

**A) and B)** Bar plots depicting fractions of genomic A) U6 (D16) and B) U6atac (D0) snRNA 3’ ends in WT and USB1 c.531delA cells (average+/-S.D., n=2 biological replicates). **C) and D)** Bar plots depicting fractions of post-transcriptionally adenylated C) U6 (D16) and D) U6atac (D0) snRNA 3’ ends in WT and USB1 c.531delA cells (average+/-S.D., n=2 biological replicates). **E) and F)** Length distribution of (A) tails at 3’ ends of U6 snRNA in E) undifferentiated hESCs or F) CD34+CD45+ hematopoietic progenitors in WT and USB1 c.531delA mutants (average+/-S.D., n=2 biological replicates).

**Fig. S4.**

**A)** Significantly enriched GO terms for DEGs in day 16 of CD34+CD45+ hematopoietic progenitors in USB1 c.531delA. **B)** Flow cytometry analysis for high SSC populations (indicative of high granularity) in WT and USB1 c.531delA cells upon neutrophil development (average+/-S.D., n=2 biological replicates). **C)** Volcano plot depicting transcriptome changes in mature blood cells (day 30) in WT and USB1 c.531delA (Gray: unchanged; Magenta: differentially expressed). **D)** Pie charts depicting splicing changes in USB1 c.531delA compared to WT cells at different stages of differentiation. Numbers indicate the total number of local splice variants in each category (black) and the percentage of differentially spliced versus unchanged (white). **E)** Venn diagram showing DEGs and differentially spliced genes in WT and USB1 c.531delA in undifferentiated hESCs (Day 0) or CD34+CD45+ hematopoietic progenitors (Day 16). **F)** KEGG pathway analysis of differentially expressed miRNAs in CD34+CD45+hematopoietic progenitors in USB1 c.531delA. **G)** Volcano plot depicting non-coding RNA changes in undifferentiated hESCs in WT and USB1 c.531delA cells (Gray: unchanged; Magenta: differentially decreased; Green: differentially increased). **H)** Representative northern blot analysis for E1 and U64 snoRNAs in undifferentiated USB1 c.531delA cells treated with DMSO or RG7834 (1µM). Relative band intensity of indicated snoRNAs normalized to 5s rRNA was shown in numbers (n=2 biological replicates). **I)** Volcano plot depicting non-coding RNA changes in CD34+CD45+ hematopoietic progenitors in WT and USB1 c.531delA cells (Gray: unchanged; Magenta: differentially decreased; Green: differentially increased).

**Fig. S5.**

**A)** Fold change of (A) tail frequency at mature ends in USB1 c.531delA versus WT hESCs (average, n=3 biological replicates). Shown are the most reduced (at least 70%) miRNAs (top 20 hits plus miR125a/b-5p) in USB1 c.531delA hESCs when compared to WT. Red bars show miRNAs with increased (A) tail frequency in USB1 c.531delA hESCs (blue dotted line equals 1). **B)** Fold change of (A) tail frequency at mature ends in USB1-KO versus WT K562 cells (average, n=3 biological replicates). Shown are the most reduced miRNAs (top 42 hits) in USB1-KO K562 when compared to WT. Red bars show miRNAs with increased (A) tail frequency in USB1-KO K562 hematopoietic cells (blue dotted line equals 1). Right panel: Knock-out of USB1 in USB1-KO K562 was confirmed by immunoblotting. Positive control: USB1_KO cells transfected with WT-USB1 plasmid. **C)** Quantification of decay rates for guide and passenger strands for miR-142, miR-199a, and miR-223 in WT and USB1 c.531delA hESCs at 0, 4, and 8hr after transcriptional shut-off with Act-D at 5µg/ml (average+/-S.D., n=3). **D)** Quantification of decay rates for miR-142-5p, miR-199a-3p, and miR-223-3p in WT and USB1-KO K562 hematopoietic cells at 0, 4, and 7hr after transcriptional shut-off with Act-D at 5µg/ml (average+/-S.D., n=3 biological replicates). **E)** Levels of guide or passenger strands of the indicated miRNAs 7hr after transcriptional shut-off with Act-D (5µg/ml) in K562 USB1-KO cells (average+/-S.D., n=3 biological replicates).

**Fig. S6.**

**A)** miRNA-seq analysis (Normalized CPM count) for guide or passenger strands of the indicated miRNAs in WT and USB1 c.531delA hESCs or WT and USB1-KO K562 cells (average+/-S.D., n=3 biological replicates). **B)** Influence of USB1 on miRNA guide and passenger strand levels. Shown are the expression levels of guide and passenger strands for miRNAs with the highest differential expression between USB1-KO and WT K562 (average, n=3 biological replicates). **C)** Gel image showing processing of 16-mer RNA substrates by wild-type USB1 or H208Q catalytic mutant. Marker is a 7-nt RNA preceding the tail in each RNA substrate. **D)** Time-course measurement of USB1’s activity on indicated RNA substrates.

**Fig. S7.**

**A)** Relative PAPD5 mRNA levels in USB1 c.531delA_shPAPD5 hESCs (average+/-S.D., n=3 biological replicates). **B)** Relative miR-125a-5p and miR-125b-5p levels in WT hESCs treated with DMSO or RG7834 (1μM) (average+/-S.D., n=2 biological replicates). **C)** CFC potential of definitive hematopoietic progenitors in WT treated with DMSO or RG7834 (0.5µM) (average+/-S.D., n=3 biological replicates). **D)** Flow cytometry analysis for CD45+CD14-CD11b+CD16+ in USB1 c.531delA treated with DMSO or RG7834 (0.5µM) upon neutrophil development (average+/-S.D., n=3 biological replicates). **E)** Bar plot depicting fractions of genomic U6 snRNA 3’ ends in USB1 c.531delA cells treated with DMSO or RG7834 (1µM) (Average+/-S.D., n=2 biological replicates). **F)** Relative levels of the indicated miRNAs in WT_miR122-5p, USB1_c.531delA_miR-122-5p and USB1 c.531delA_PC miRs hESCs (average+/-S.D., n=2 technical replicates). **G)** Relative levels of the indicated miRNAs in definitive hematopoietic progenitors in WT cells treated with miRNA inhibitors specifically targeting miR-125a-5p, 142-5p, 199a-3p, and 223-3p (average+/-S.D., n=2 biological replicates).

| **Gene** | **Forward sequence** | **Reverse sequence** |
| --- | --- | --- |
| **USB1** | CCCTTCCCAGGCAGAGATTT | CTCCTCCTTGGCTTCATATGGT |
| **GAPDH** | AGAAGGCTGGGGCTCATTTG | AGGGGCCATCACAGTCTTC |
| **POU5F1** | TGCAGAAAGAACTCGAGCAA | ACACTCGGACCACATCCTTC |
| **NANOG** | TGAACCTCAGCTACAAACAG | TGGTGGTAGGAAGAGTAAAG |
| **T** | TGTCCCAGGTGGCTTACAGATGAA | GGTGTGCCAAAGTTGCCAATACAC |
| **CD34** | GCCTTGCAACATCTCCCACT | CCTTCTTAAACTCCGCACAGC |
| **GATA2** | CTCGTTCCTGTTCAGAAGGC | AATTTGCACAACAGGTGCCG |
| **PAPD5** | AGGAACTACAACCAGGGAGTC | ACCTCCATCCGCATCTTCT |
| **RUNX1** | CTACCGCAGCCATGAAGAA | GACAGTGATGGTCAGAGTGAAG |
| **RUNX2** | CATCACTGTCCTTTGGGAGTAG | ATGTCAAAGGCTGTCTGTAGG |
| **CEBPA** | GATAACCTTGTGCCTTGGAAATG | GAGGCAGGAAACCTCCAAATA |
| **CEBPE** | CAGCTTCTCTCCGATCTCTTTG | GTCAGGCGGCAAGTAGTG |

**Table S1**

qRT-PCR primer sequences used.

| **LNA probes** | **Sequence** |
| --- | --- |
| **miR-125a-5p** | TCACAGGTTAAAGGGTCTGAGGGA |
| **miR-125b-5p** | TCACAAGTTAGGGTCTCAGG |
| **E1 snoRNA** | CGTTGTGGAAAGGGACTTGTAC |
| **U64 snoRNA** | GGTGAAGCCAAGTGCAACTATGC |
| **5s RNA** | CGTTCAGGGTGGTATGGCCG |

**Table S2**

LNA probes used.

| **Oligos** | **Sequence** |
| --- | --- |
| **miR-125a-native** | UCCCUGAGACCCUUUAACCUGUGA |
| **miR-125a-oA** | UCCCUGAGACCCUUUAACCUGUGAAAAAA |
| **miR-125a-oU** | UCCCUGAGACCCUUUAACCUGUGAUUUUU |
| **miR-125a-oUA** | UCCCUGAGACCCUUUAACCUGUGAUUAAA |
| **miR-125a-oAU** | UCCCUGAGACCCUUUAACCUGUGAAAUUU |
| **U6-oU** | AAUUCGUGAAGCGUUCCAUAUUUUU |
| **U6-oA** | AAUUCGUGAAGCGUUCCAUAUUUUUAAAAAAA |
| **U6-oUA** | AAUUCGUGAAGCGUUCCAUAUUUUUAAUAAUAAUAAUAA |
| **16mer-native** | CCUUUCCCCUUUCCGC |
| **16mer-oA** | CCUUUCCAAAAAAAAA |
| **16mer-oU** | CCUUUCCUUUUUUUUU |
| **16mer-oUA** | CCUUUCCUUUUAAAAA |
| **16mer-oAU** | CCUUUCCAAAAUUUUU |

**Table S3**

RNA oligo sequences used.
